# Association between maternal gestational weight gain and preterm birth according to body mass index and maternal age in Quzhou, China

**DOI:** 10.1038/s41598-020-72949-w

**Published:** 2020-09-28

**Authors:** Ying Hu, Qi Wu, Luyang Han, Yuqing Zou, Die Hong, Jia Liu, Yuying Zhu, Qiumin Zhu, Danqing Chen, Lu Qi, Zhaoxia Liang

**Affiliations:** 1 Obstetrical Department, Women’s Hospital, School of Medicine, Zhejiang University, Hangzhou, 310006 China; 2 Quzhou Maternal and Child Health Hospital, Quzhou Maternal and Child Medical Association, Zhejiang University, Hangzhou, China; 3 Department of Epidemiology, School of Public Health and Tropical Medicine, Tulane University, New Orleans, LA USA

**Keywords:** Diseases, Health care, Medical research, Risk factors

## Abstract

The aim of this study is to investigate the association between maternal gestational weight gain (GWG) and preterm birth according to pre-pregnancy body mass index (BMI) and maternal age. We did a cohort, hospital-based study in Quzhou, South China, from 1 Jan 2018 to 30 June 2019. We selected 4274 singleton live births in our analysis, 315 (7.4%) of which were preterm births. In the overall population, excess GWG was significantly associated with a decreased risk of preterm birth compared with adequate GWG (adjusted OR 0.81 [95% CI 0.72–0.91]), and the risk varied by increasing maternal age and pre-pregnancy BMI. Interestingly, underweight women who older than 35 years with excess GWG had significantly increased odds of preterm birth compared with adequate GWG in underweight women aged 20–29 years (2.26 [1.06–4.85]) and normal weight women older than 35 years (2.23 [1.13–4.39]). Additionally, low GWG was positively and significantly associated with preterm birth overall (1.92 [1.47–2.50]). Among normal weight women category, compared with adequate GWG women aged 20–29 years did, those older than 20 years with low GWG, had significantly higher odds of preterm birth, which increased with maternal age (1.80 [1.16–2.79] in 20–29 years, 2.19 [1.23–3.91] in 30–34 years, 3.30 [1.68–6.46] in ≫ 35 years). In conclusion, maternal GWG was significantly associated with the risk of preterm birth, but the risk varied by pre-pregnancy BMI and maternal age.

## Introduction

Preterm birth is one of common adverse perinatal outcomes that affects approximately 11% of births worldwide^1^. It is reported that the incidence of preterm birth in China increased significantly recently, from 5 to 10%, and 1.5 million preterm infants are born every year. As the well‐known risk factor of perinatal and neonatal morbidity, preterm birth is also considered as the cause leading to long-term adverse outcomes in children^2^. Therefore, it is imperative to identify risk factors for preterm birth and explore preventive measures to reduce it.

Gestational weight gain (GWG), whether too much or too little, is associated with adverse maternal and infant outcomes^3,4^. With excess GWG, pregnant women are more likely to experience gestational diabetes mellitus and macrosomia, which can contribute to preterm birth^5,6^. Meanwhile low GWG increases the risk of small for gestational age, low birth weight, as well as preterm birth^7^. As early as 1990, the Institute of Medicine (IOM) was the first to recommend guidelines for GWG. With the growing knowledge, the IOM revised its recommendations for GWG in single pregnancy women in 2009. However, they do not apply to areas in the world where women are much shorter or thinner than American women or have insufficient obstetric services^8^.

Many studies have used the IOM recommendations as reference to investigate the relationship between GWG and pregnancy outcomes^9–11^. In 2018, China recommended guidelines for GWG in single pregnancy women, which were in line with IOM guidelines. However, the association between GWG and preterm birth is still controversial and inconclusive. Although low GWG was a risk factor for preterm birth in some studies, in others positive or inverse associations between excess GWG and preterm birth were noted^10^. Otherwise, pre-pregnancy BMI might play an important role in the relationship between GWG and preterm birth^12,13^. Similarly, the function of maternal age, too, cannot be overlooked^8,14^. But almost all of those studies were carried out in industrialized countries. Only a few studies have evaluated this issue in non-industrialized countries, including China.

In this cohort study, we aimed to examine the association between GWG and preterm birth according to pre-pregnancy BMI and maternal age in China. Promoting appropriate weight gain advice and prenatal care to mothers before and during pregnancy might reduce the burden of preterm birth.

## Results

### Prevalence of preterm birth in the study population

**Figure 1:**
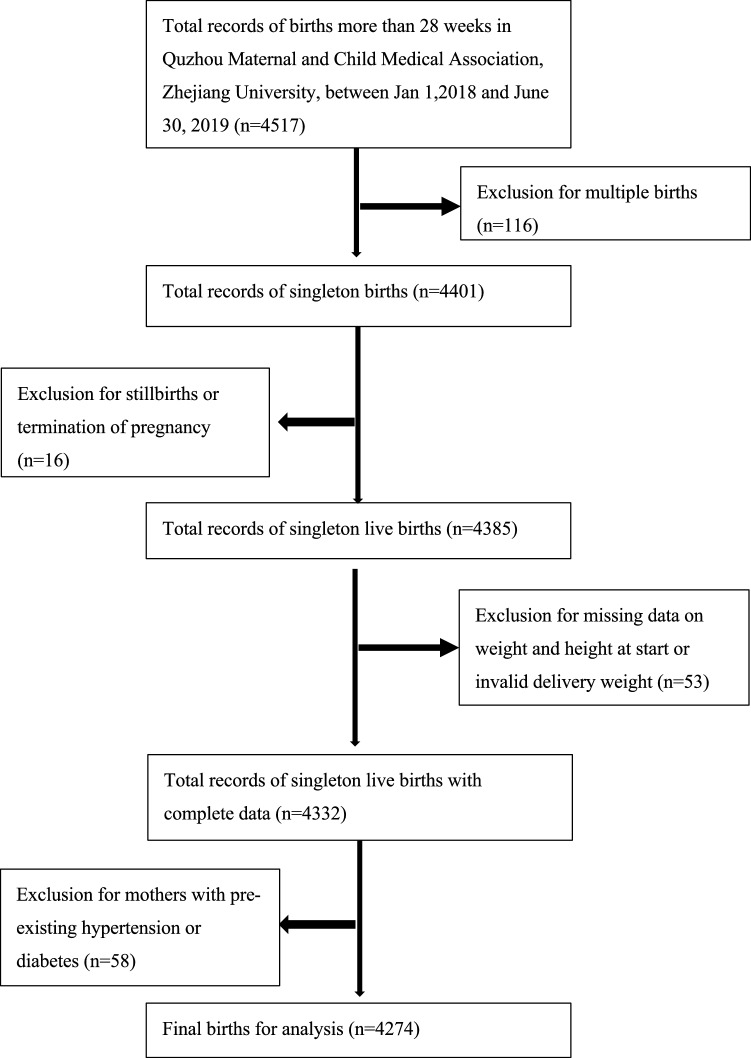
Flow chart of participant selection.

Our study finally enrolled 4274 women of live singleton births with complete data (Fig. 1), of which 315 (7.4%) were preterm births. The proportion of births that were preterm was significantly higher among women with higher BMI than those with lower BMI (p = 0.020). Women with low GWG had higher prevalence of preterm birth, while those with excess GWG had lower, comparing with adequate GWG women (p = 0.000). Women younger than 20 years or older than 35 years were more likely to cause preterm birth (p = 0.000), similar results were found in women with previous history of preterm birth (p = 0.000). Additionally, less education (p = 0.025) and lack of prenatal care (p = 0.000) might be considered as potential factors for preterm birth. Higher proportion of preterm birth also could be found in women who were conceived by ART (p = 0.038), had pregnancy-induced hypertension, (p = 0.000), delivered male infants (p = 0.012), than among those who did not (Table 1). The prevalence of preterm birth in each GWG category by population characteristics is depicted in Table S1.

**Table 1:** Prevalence of preterm birth in the study population.

	N	Preterm birth	P value	Moderately preterm birth	Very preterm birth	Extremely preterm birth
Overall	4274	315 (7.4%)		250 (5.8%)	41 (1.0%)	24 (0.6%)
**Age**			0.000			
< 20	47	8 (17.0%)		4 (8.5%)	0	4 (8.5%)
20–29	2329	145 (6.2%)		117 (5.0%)	20 (0.9%)	8 (0.3%)
30–34	1218	89 (7.3%)		71 (5.8%)	12 (9.9%)	6 (0.5%)
$\gg$ 35	680	73 (10.7%)		58 (8.5%)	9 (1.3%)	6 (0.9%)
**Education level**			0.025			
Primary	96	13 (13.5%)		9 (9.4%)	1 (1.0%)	3 (3.1%)
Secondary	2089	167 (8.0%)		126 (6.0%)	25 (1.2%)	16 (0.8%)
College	2008	131 (6.5%)		112 (5.6%)	15 (0.7%)	4 (0.2%)
Post-graduate	81	4 (4.9%)		3 (3.7%)	0	1 (1.2%)
**Pre-pregnancy BMI (kg/m**^**2**^**)**			0.020			
Underweight < 18.5	695	35 (5.0%)		28 (4.0%)	4 (0.6%)	3 (0.4%)
Normal 18.5 $\ll$ BMI $\ll$ 24.9	3133	238 (7.6%)		191 (6.1%)	30 (1.0%)	17 (0.5%)
Overweight 25 $\ll$ BMI $\ll$ 29.9	402	36 (9.0%)		25 (6.2%)	7 (1.7%)	4 (1.0%)
Obese $\gg$ 30	44	6 (13.6%)		6 (13.6%)	0	0
**Total GWG**			0.000			
Low	965	114 (11.8%)		83 (8.6%)	18 (1.9%)	13 (1.3%)
Adequate	2135	150 (7.0%)		118 (5.5%)	21 (1.0%)	11 (0.5%)
Excess	1174	51 (4.3%)		49 (4.2%)	2 (0.2%)	0
**Gravidity (before current pregnancy)**			0.055			
0	1208	81 (6.7%)		66 (5.5%)	11 (0.9%)	4 (0.3%)
1–2	2054	142 (6.9%)		104 (5.1%)	23 (1.1%)	15 (0.7%)
$\gg$ 3	1012	92 (9.1%)		80 (7.9%)	7 (0.7%)	5 (0.5%)
**Parity (before current pregnancy)**			0.161			
Nullipara	2089	142 (6.8%)		112 (5.4%)	20 (1.0%)	10 (0.5%)
Multipara	2185	173 (7.9%)		138 (6.3%)	21 (1.0%)	14 (0.6%)
**Previous history of preterm birth**			0.000			
Yes	119	28 (23.5%)		21 (17.6%)	3 (2.5%)	4 (3.4%)
No	2066	145 (7.0%)		117 (5.7%)	18 (0.9%)	10 (0.5%)
Nullipara	2089	142 (6.8%)		112 (5.4%)	20 (1.0%)	10 (0.5%)
**Mode of conception**			0.038			
Nature conceived	4161	301 (7.2%)		240 (5.8%)	39 (0.9%)	22 (0.5%)
ART	113	14 (12.4%)		10 (8.8%)	2 (1.8%)	2 (1.8%)
**Prenatal care**			0.000			
None	25	4 (16.0%)		2 (8.0%)	1 (4.3%)	1 (4.0%)
Our hospital	3185	199 (6.2%)		172 (5.4%)	16 (0.5%)	11 (0.3%)
Other hospitals	1064	112 (10.5%)		76 (7.1%)	24 (2.3%)	12 (1.1%)
**Timing of initiation of prenatal care**			0.937			
1st–3rd month	3833	279 (7.3%)		222 (5.8%)	37 (1.0%)	20 (0.5%)
4th–6th month	393	30 (7.6%)		24 (6.1%)	3 (0.8%)	3 (0.8%)
7th to final month	23	2 (8.7%)		2 (8.7%)	0	0
None	25	4 (16.0%)		2 (8.0%)	1 (4.0%)	1 (4.0%)
Pregnancy-induced hypertension (including preeclampsia)	126	22 (17.5%)	0.000	16 (12.7%)	5 (4.0%)	1 (0.8%)
Gestational diabetes mellitus	922	70 (7.6%)	0.771	53 (5.7%)	12 (1.3%)	5 (0.5%)
**Infant sex**			0.012			
Male	2220	185 (8.3%)		145 (6.5%)	26 (1.2%)	14 (0.6%)
Female	2054	130 (6.3%)		105 (5.1%)	15 (0.7%)	10 (0.5%)

Data were n/N (%). Preterm birth was defined as delivery occurring before 37 weeks of gestation. Moderately preterm births were those at 34–36 + 6 weeks’ gestation. Very preterm births were those at 32–33 + 6 weeks’ gestation. Extremely preterm births were those at 28–31 + 6 weeks’ gestation.

### Association between GWG and preterm birth

In the overall population, mothers who with low GWG had a significantly increased risk of preterm birth compared with adequate GWG (adjusted OR 1.92 [95% CI 1·47–2.50]), while excess GWG was negatively and significantly associated with preterm birth (adjusted OR 0.81 [95% CI 0.72–0.91]). In the subsequent stratified analyses, pre-pregnancy BMI or maternal age modified the associations between GWG and preterm birth on the basis of the IOM recommendations. It was indicated that the positive relationship between low GWG and preterm birth consistently across maternal age groups and pre-pregnancy BMI groups, in which the adjusted ORs of preterm birth varied by increasing maternal age and pre-pregnancy BMI. Similarly, the significant inverse association between excess GWG and preterm birth also could be partly found in different subgroups. Interestingly, women who were older than 35 years or underweight tended to have positive relationships between excess GWG and preterm birth, although it was not statistically significant. (Table 2).

**Table 2:** ORs for the association between GWG and preterm birth.

	Low GWG			Adequate GWG		Excess GWG		
	n1/n2	Crude OR (95% CI)	Adjusted OR (95% CI)	n1/n2	OR (95% CI)	n1/n2	Crude OR (95% CI)	Adjusted OR (95% CI)
Overall	114/851	1.77 (1.37–2.29)*	1.92 (1.47–2.50) *	150/1985	Ref	51/1123	0.60 (0.43–0.83)*	0.81 (0.72–0.91) *
**Age groups**^**a**^								
< 20	4/9	1.44 (0.28–7.34)	4.16 (0.29–59.91)	4/13	Ref	0/17	/	/
20–29	49/431	1.62 (1.11–2.36)*	1.76 (1.20–2.59) *	76/1085	Ref	20/668	0.43 (0.26–0.71) *	0.72 (0.60–0.86) *
30–34	34/249	2.11 (1.30–3.43)*	2.44 (1.45–4.12) *	38/588	Ref	17/292	0.90 (0.50–1.62)	0.87 (0.70–1.08)
$\gg$ 35	27/162	1.56 (0.90–2.69)	1.79 (1.00–3.12) *	32/299	Ref	14/146	0.90 (0.46–1.73)	1.01 (0.80–1.28)
**Pre-pregnancy BMI**^**b**^								
Underweight	15/175	2.19 (1.03–4.63)*	2.26 (1.03–4.96) *	14/357	Ref	6/128	1.20 (0.45–3.18)	1.11 (0.78–1.57)
Normal	93/646	1.84 (1.38–2.45)*	1.94 (1.44–2.62) *	117/1492	Ref	28/757	0.47 (0.31–0.72)*	0.78 (0.68–0.90) *
Overweight and obese	6/30	1.43 (0.53–3.89)	1.46 (0.51–4.21)	19/136	Ref	17/238	0.51 (0.26–1.02)	0.77 (0.60–0.99) *

n1/n2 means number of preterm/term births. Adjusted for maternal delivery age, education level, parity, gravidity, pre-pregnancy BMI, previous premature delivery, mode of conception, prenatal care, timing of initiation of prenatal care, pregnancy-induced hypertension, gestational diabetes mellitus and sex of infant.

*OR* odds ratio, *CI* confidence interval, *Ref* reference.

*p < 0.05.

^a^Maternal delivery age was not included in this model.

^b^Pre-pregnancy BMI was not included in this model.

### Association between GWG and different degrees of preterm birth

The associations between GWG and moderately, very, and extremely preterm births are shown in Table 3. Mothers who with excess GWG (adjusted OR 0.88 [95% CI 0.78–0.99]) had a significantly decreased risk of moderately preterm birth compared with adequate GWG. Stratified analysis showed that the lowest risk group was the 20–29 years age group (adjusted OR 0.75 [95% CI 0.63–0.91]) or the normal pre-pregnancy BMI group (adjusted OR 0.84 [95% CI 0.72–0.97]). Compared with adequate GWG, low GWG was positively and significantly associated with moderately preterm birth overall (adjusted OR 1·79 [95% CI 1.33–2.42]). And the risk increased with maternal age in women older than 20 years, but stratified analysis also showed a potential trend that the highest risk group might be the age group younger than 20 years. Meanwhile, it also showed that the risk of moderately preterm birth in women with low GWG was 1.73 times greater than those with adequate GWG among normal weight groups.

**Table3:** Adjusted ORs (95% CI) for the association between GWG and different groups of preterm birth.

	Low GWG			Adequate GWG	Excess GWG		
	Moderately preterm birth	Very preterm birth	Extremely preterm birth	OR	Moderately preterm birth	Very preterm birth	Extremely preterm birth
Overall	1.79 (1.33–2.42) *	2.10 (1.09–4.05) *	3.10 (1.32–7.28) *	Ref	0.88 (0.78–0.99) *	0.49 (0.30–0.82) *	/
**Age groups**^a^							
< 20	13.18 (0.11–1562)	/	2.95 (0.03–315)	Ref	/	/	/
20–29	1.65 (1.07–2.54) *	3.10 (1.13–8.51) *	1.39 (0.31–6.31)	Ref	0.75 (0.63–0.91) *	0.59 (0.34–1.05)	/
30–34	1.93 (1.06–3.52) *	3.03 (0.91–10.07)	15.98 (1.60–159) *	Ref	0.92 (0.74–1.15)	/	/
$\gg$ 35	2.06 (1.08–3.93) *	0.77 (0.14–4.23)	2.67 (0.30–24.18)	Ref	1.17 (0.91–1.49)	/	/
**Pre-pregnancy BMI**^**b**^							
Underweight	1.80 (0.73–4.43)	6.78 (0.56–81.82)	6.27 (0.37–107.44)	Ref	1.16 (0.82–1.66)	/	/
Normal	1.73 (1.24–2.42) *	2.44 (1.16–5.16) *	4.18 (1.48–11.82) *	Ref	0.84 (0.72–0.97) *	0.16 (0.00–6.33)	0.18 (0.00–14.6)
Overweight and obese	2.98 (0.93–9.52)	/	/	Ref	0.91 (0.68–1.21)	0.67 (0.40–1.12)	0.54 (0.26–1.12)

Adjusted for maternal delivery age, education level, parity, gravidity, pre-pregnancy BMI, previous premature delivery, mode of conception, prenatal care, timing of initiation of prenatal care, pregnancy-induced hypertension, gestational diabetes mellitus, and sex of infant.

*OR* odds ratio, *CI* confidence interval, *Ref* reference.

*p < 0.05.

^a^Maternal delivery age was not included in this model.

^b^Pre-pregnancy BMI was not included in this model.

As for very preterm birth, mothers with excess GWG (adjusted OR 0.49 [95% CI 0.30–0.82]) also had a significantly decreased risk, and who with low GWG had increased risk (adjusted OR 2.10 [95% CI 1·09–4.05]). Moreover, due to small sample size of extremely preterm birth, we didn’t have too much results in stratified analysis. However, it was also explored that the risk of low GWG to preterm birth increased with its severity, especially in women with normal weight. The opposite results were found in women with excess GWG.

### Joint association of maternal age and GWG with risk of preterm birth in different pre-pregnancy BMI groups

**Figure 2:**
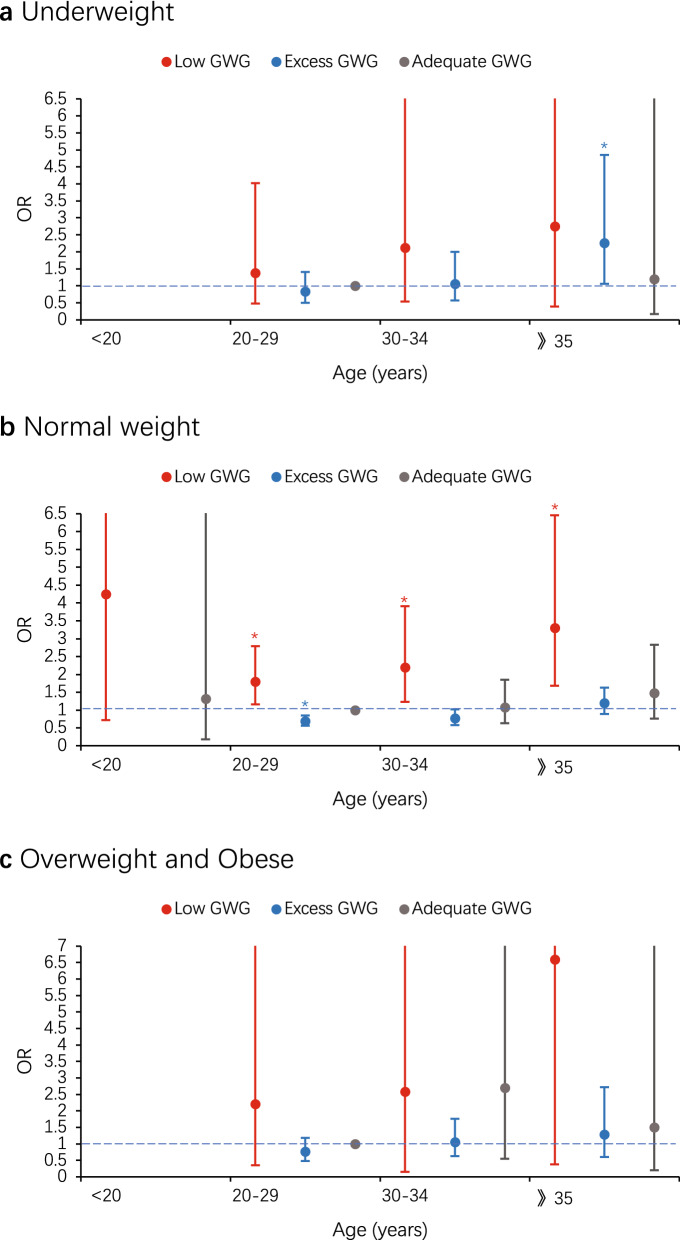
Joint association of maternal age and GWG with risk of preterm birth in underweight (**a**), normal (**b**), overweight and obese (**c**). Education level, parity, gravidity, previous premature delivery, mode of conception, prenatal care, timing of initiation of prenatal care, pregnancy-induced hypertension, gestational diabetes mellitus and sex of infant were adjusted for in models. Women aged 20–29 years who had an adequate GWG were the reference group. Error bars present 95% CI. *OR* odds ratio. *p < 0.05.

To further explore the relations between GWG and preterm birth, we examined joint effects of maternal age with GWG on the risk of preterm birth (Table 4). Among normal weight women category, compared with adequate GWG women aged 20–29 years did, those older than 20 years with low GWG, had significantly higher odds of preterm birth, which increased with maternal age. But similar result was only found in excess GWG women aged 20–29 years, which was regard as negative factors (adjusted OR 0.69 [95% CI 0.56–0.85]) (Fig. 2b). There were no significant results observed among other pre-pregnancy BMI category (Fig. 2c), except for underweight women who older than 35 years with excess GWG. Those women had significantly increased odds of preterm birth compared with adequate GWG women aged 20–29 years did (adjusted OR 2.26 [95% CI 1.06–4.85]), which was inconsistent with above results (Fig. 2a).

**Table 4:** Joint association of maternal age and GWG with risk of preterm birth in different pre-pregnancy BMI groups.

Pre-pregnancy BMI	Low GWG			Adequate GWG			Excess GWG		
	n1/n2	Crude OR (95% CI)	Adjusted OR (95% CI)	n1/n2	Crude OR (95% CI)	Adjusted OR (95% CI)	n1/n2	Crude OR (95% CI)	Adjusted OR (95% CI)
**Underweight**									
< 20	2/1	50.6 (4.23–605.58)	/	1/1	25.3 (1.47–434.30)	14.7 (0.47–464.53)	0/2	/	/
20–29	6/111	1.37 (0.49–3.86)	1.39 (0.48–4.03)	10/253	Ref		2/101	0.50 (0.11–2.33)	0.84 (0.50–1.41)
30–34	5/40	3.16 (1.03–9.73)	2.13 (0.54–8.43)	1/79	0.32 (0.04–2.54)	/	2/22	2.30 (0.47–11.16)	1.06 (0.57–2.00)
$\gg$ 35	2/23	2.20 (0.45–10.65)	2.75 (0.39–19.22)	2/24	2.11 (0.44–10.18)	1.20 (0.17–8.38)	2/3	16.87 (2.53–112.49)*	2.26 (1.06–4.85) *
**Normal**									
< 20	2/8	3.18 (0.66–15.29)	4.25 (0.72–24.94)	3/11	3.46 (0.94–12.75)	1.32 (0.18–9.70)	0/10	/	/
20–29	40/306	1.66 (1.09–2.53)*	1.80 (1.16–2.79) *	60/762	Ref		12/451	0.34 (0.18–0.64)*	0.69 (0.56–0.85) *
30–34	28/200	1.78 (1.11–2.86)*	2.19 (1.23–3.91) *	30/467	0.82 (0.52–1.28)	1.08 (0.63–1.85)	7/207	0.43 (0.19–0.95)*	0.77 (0.58–1.02)
$\gg$ 35	23/132	2.21 (1.32–3.70)*	3.30 (1.68–6.46) *	24/252	1.21 (0.74–1.98)	1.47 (0.76–2.83)	9/89	1.28 (0.62–2.68)	1.20 (0.89–1.63)
**Overweight and obese**									
< 20	0/0	/	/	0/1	/	/	0/5	/	/
20–29	3/14	2.50 (0.56–11.20)	2.21 (0.35–13.89)	6/70	Ref		6/116	0.60 (0.19–1.94)	0.76 (0.48–1.18)
30–34	1/9	1.30 (0.14–12.03)	2.58 (0.15–43.36)	7/42	1.94 (0.61–6.18)	2.70 (0.55–13.25)	8/63	1.48 (0.49–4.50)	1.05 (0.63–1.76)
$\gg$**35**	2/7	3.33 (0.56–19.75)	6.59 (0.38–114.58)	6/23	3.04 (0.89–10.37)	1.50 (0.20–11.23)	3/54	0.65 (0.16–2.71)	1.28 (0.60–2.72)

n1/n2 means number of preterm/term births. Adjusted for education level, parity, gravidity, previous premature delivery, mode of conception, prenatal care, timing of initiation of prenatal care, pregnancy-induced hypertension, gestational diabetes mellitus and sex of infant. Women aged 20–29 years who had an adequate GWG were the reference group.

*OR* odds ratio, *CI* confidence interval.

*p < 0.05.

### Joint association of pre-pregnancy BMI and GWG with risk of preterm birth among women in different age groups

**Figure 3:**
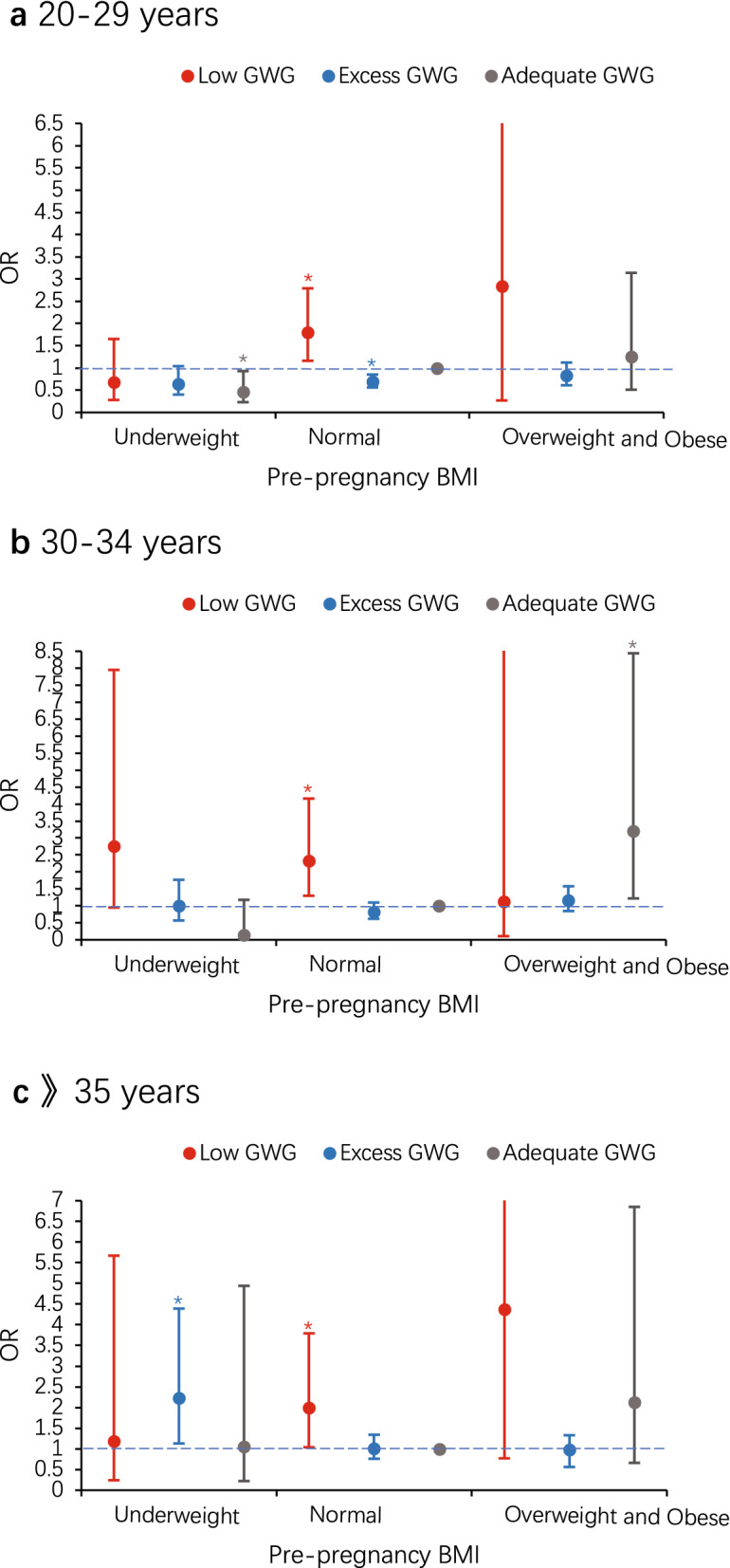
Joint association of pre-pregnancy BMI and GWG with risk of preterm birth among women aged 20–29 years (**a**), 30–34 years (**b**) and 35 years or older (**c**). Education level, parity, gravidity, previous premature delivery, mode of conception, prenatal care, timing of initiation of prenatal care, pregnancy-induced hypertension, gestational diabetes mellitus and sex of infant were adjusted for in models. The reference group were women who had a normal pre-pregnancy BMI and an adequate GWG. Error bars present 95% CI. *OR* odds ratio. *p < 0.05.

We also examined joint effects of pre-pregnancy BMI with GWG on the risk of preterm birth (Table 5). In women category aged 20–29 years, adequate GWG was regard as a protective factor for preterm delivery in those classified as underweight, similar in normal weight women with excess GWG (Fig. 3a). In women category aged 30–34 years, those classified as overweight and obese had the highest risk of preterm delivery within adequate or excess GWG groups, however those classified as underweight tended to have the highest risk of preterm delivery within low GWG group (Fig. 3b). Interestingly, we observed the opposite result again in women category older than 35 years and underweight, who had the significantly highest risk of preterm delivery within excess GWG group (Fig. 3c).

**Table 5:** Joint association of pre-pregnancy BMI and GWG with risk of preterm birth among women in different age groups.

Age groups	Low GWG			Adequate GWG			Excess GWG		
	n1/n2	Crude OR (95% CI)	Adjusted OR (95% CI)	n1/n2	Crude OR (95% CI)	Adjusted OR (95% CI)	n1/n2	Crude OR (95% CI)	Adjusted OR (95% CI)
**< 20**									
Underweight	2/1	7.33 (0.48–111.19)	5.26 (0.17–159.79)	1/1	3.67 (0.17–77.55)	/	0/2	/	/
Normal	2/8	0.92 (0.12–6.83)	/	3/11	Ref		0/10	/	/
Overweight and obese	0/0	/	/	0/1	/	/	0/5	/	/
**20–29**									
Underweight	6/111	0.69 (0.29–1.63)	0.68 (0.28–1.65)	10/253	0.50 (0.25–1.00)*	0.46 (0.23–0.93)*	2/101	0.25 (0.06–1.04)	0.64 (0.40–1.04)
Normal	40/306	1.66 (1.09–2.53)*	1.80 (1.16–2.79)*	60/762	Ref		12/451	0.34 (0.18–0.64)*	0.69 (0.56–0.85)*
Overweight and obese	3/14	2.72 (0.76–9.73)	2.84 (0.77–10.50)	6/70	1.09 (0.45–2.61)	1.26 (0.51–3.14)	6/116	0.66 (0.28–1.56)	0.83 (0.61–1.12)
**30–34**									
Underweight	5/40	1.95 (0.72–5.29)	2.75 (0.95–7.95)	1/79	0.20 (0.03–1.47)	0.14 (0.02–1.18)	2/22	1.42 (0.32–6.30)	1.01 (0.57–1.77)
Normal	28/200	2.18 (1.27–3.74)*	2.32 (1.30–4.16)*	30/467	Ref		7/207	0.53 (0.23–1.22)	0.83 (0.62–1.10)
Overweight and obese	1/9	1.73 (0.21–14.11)	1.13 (0.11–11.32)	7/42	2.59 (1.08–6.26)*	3.21 (1.22–8.44)*	8/63	1.98 (0.87–4.50)	1.16 (0.85–1.58)
$\gg$** 35**									
Underweight	2/23	0.91 (0.20–4.11)	1.18 (0.24–5.67)	2/24	0.88 (0.20–3.93)	1.05 (0.22–4.94)	2/3	7.00 (1.11–43.97)	2.23 (1.13–4.39)*
Normal	23/132	1.83 (1.00–3.37)	1.99 (1.04–3.79)*	24/252	Ref		9/89	1.06 (0.48–2.37)	1.01 (0.76–1.34)
Overweight and obese	2/7	3.00 (0.59–15.26)	4.38 (0.77–24.73)	6/23	2.74 (1.02–7.38)	2.13 (0.66–6.85)	3/54	0.58 (0.17–2.01)	0.98 (0.56–1.33)

n1/n2 means number of preterm/term births. Adjusted for education level, parity, gravidity, previous premature delivery, mode of conception, prenatal care, timing of initiation of prenatal care, pregnancy-induced hypertension, gestational diabetes mellitus and sex of infant. Women who had both a normal pre-pregnancy BMI and an adequate GWG were the reference group.

*OR* odds ratio, *CI* confidence interval.

*p < 0.05.

## Discussion

The increasing frequency of preterm birth worldwide makes it a critical endpoint to consider in relationship between GWG and preterm birth, which was inconsistent and inconclusive in previous studies. In the 1990s, quite limited literatures pointed out low GWG increased the risk of preterm birth. Some other previous studies showed an inverse association between excess GWG and preterm birth^2,9^. In those studies, women with lower GWG were at higher risk of preterm birth than higher GWG. However, a review including 12 studies showed that the risk of preterm birth increased consistently in both the highest and lowest GWG categories^15^. A potential positive association between excess GWG and preterm birth was also reported in recent study^10^. It is interesting to note that these studies used different definitions of high and low GWG, as well as different analytic methods to characterize the association between GWG and preterm birth, which might lead to the controversial results.

In our study of 4274 mother–child pairs, we noted that women with low GWG have a significantly increased risk of preterm birth compared to women with adequate GWG. Additionally, we found women aged 30–34 and underweight women had highest risk of preterm birth if they didn't gain enough weight during pregnancy. Partially inconsistent with previous studies^7,16,17^, we also found decreased risk of preterm birth in women with excess GWG, however risk increased once those women were older than 35 years and had lower pre-pregnancy BMI, which indicated the association between GWG and preterm birth varied by maternal age and pre-pregnancy BMI. Our findings provide a comprehensive review of the association between GWG and preterm birth for women in South China for the first time and suggest that the differing maternal age and pre-pregnancy BMI might contributed to the inconsistent findings.

The effect of maternal age on the association between GWG and preterm birth suggests that age may play an important role in the potential mechanism of GWG on preterm birth. In this study, we found excess GWG could decrease the incidence of preterm birth in general, but opposite result among women aged 35 years or older. Actually, women with advanced maternal age was considered to have higher risk of preterm birth^14,18^. In these women, excess GWG could lead to pregnancy complications more likely, such as gestational diabetes mellitus and gestational hypertension, which is why the risk of preterm birth is increased^5^. Additionally, excess GWG also was associated with alterations in maternal metabolism and placental microenvironment, which was proposed as a possible pathogenesis in preterm birth^19^. Unfortunately, there were no significantly results among women younger than 20 years old in our study due to small sample size, but we cannot ignore this group. Adolescent (females younger than 20 years old) pregnancy was also showed to be associated with increased risk of preterm birth^8,20^. And Howie et al. reported that younger adolescents have an increased likelihood for greater GWG compared to older women in a retrospective review^21^. However, the relationship between GWG and preterm birth in adolescents is unclear. Only several studies showed an inverse association between pre-pregnancy obesity and preterm birth among them^22–24^. Therefore, further research is necessary among adolescents to establish the specific GWG guidelines and explore the underlying mechanisms.

The association between GWG and preterm birth was inconsistent in different BMI categories, although some previous studies considered GWG as a mediator in the association between pre-pregnancy BMI and preterm birth, rather than a confounder^25^. The association between GWG and preterm birth was modified by pre-pregnancy BMI in 5 studies in the review including 12 studies mentioned above^15^. The findings of these studies consistently showed a stronger effect of low GWG on preterm birth among underweight women. Similarly, our results also showed the magnitude of increased risk associated with low GWG diminished as pre-pregnancy BMI increased. And the risk of low GWG to preterm birth increased with maternal age, especially in underweight populations. By contrast with previous studies that showed excess GWG tended to decrease risk of preterm birth in underweight women^4^, our results indicated that the negative association between excess GWG and preterm birth was more significant among normal weight populations. Additionally, we also found that the protective effect of excess GWG to preterm birth was appeared in more age groups among normal weight women than among other pre-pregnancy BMI. Yet, we did not advocate excess GWG as a preventive method against preterm birth, as other adverse effects on perinatal and neonatal outcomes that excess GWG should not be ignored^12,26^. The tailored recommendations for pregnancy according to different pre-pregnancy BMI groups are needed to stratify the risk of preterm birth accurately.

Although not clearly elucidated, at least five possible primary pathogenic mechanisms have been postulated for preterm birth, including: activation of the maternal or fetal hypothalamic–pituitary–adrenal (HPA) axis, uteroplacental thrombosis and intrauterine vascular lesions, amniochorionic-decidual or systemic inflammation, cervical insufficiency and pathologic distention of the myometrium. There was not enough evidence so far to show a causal relationship between GWG and preterm birth, because no studies showed direct link of GWG to pathways mentioned above. Although there are intriguing data linking nutrient deficiencies to activation of the maternal or fetal HPA axis^27^, as well as increased oxidative stress and/or altered immune functions^28^, important interactions remain unanswered. However, understanding the disparities in the effects of GWG on preterm birth among women at different ages and in different pre-pregnancy BMI groups could help to identify novel pathways in the pathogenesis of preterm birth.

Our study has several limitations. First, the sample size is not large enough. Enlarging the samples is necessary for the following study. Second, this is a single-center study. Although our research center is the largest in Quzhou, it is also very important to combine with other research centers to make our findings more applicable to all populations. Third, our research did not deeply explore each subtype of preterm birth, the special reason of which may suggest its underlying pathogenesis. Fourth, while many potential confounders have been adjusted, there may still be some factors such as nutrition and supplement intake of the mother influencing the results. However, the basic nutritional status of pregnant women might be reflected in pre-pregnancy BMI, and the nutritional intake during pregnancy or the supplement intake of the mother might be partially reflected in the GWG.

In conclusion, the association between GWG and risk of preterm birth differs according to maternal age and pre-pregnancy BMI. Although inappropriate age and pre-pregnancy BMI are recognized as risk factors for pregnancy outcomes in precious studies, targeted recommendations in different clinical applications are actually needed. Obstetricians should take maternal age and pre-pregnancy BMI into account when they manage weight gain in pregnant women, so as to reduce the occurrence of preterm birth. The same is true of risk prediction models for preterm births. Otherwise, investigations to explore the underlying mechanisms for changing associations between maternal GWG and risk of preterm birth cannot be ignored in the future.

## Methods

### Study design and population

The cohort study of mother–child pairs was conducted in Quzhou, a middle-underdeveloped city in south China. We enrolled women with single pregnancy who delivered live infants at Quzhou Maternal and Child Medical Association, Zhejiang University, which is the largest obstetric center in Quzhou city, between Jan 1, 2018 and June 30, 2019. Clinical variables were abstracted after enrolment of the cases from the medical records. The information was collected by study staffs and reviewed by a medical doctor using a standard data-abstraction form. This study was approved by the Human Ethics committee at Quzhou Maternal and Child Health Hospital (Quzhou Maternal and Child Medical Association, Zhejiang University), and written informed consent was obtained from each participant. The methods were performed in accordance with the relevant guidelines and regulations.

In this study, we included all mothers who delivered a live singleton after more than 28 gestational weeks and available for GWG and pre-pregnancy BMI. The main reason for including only singleton live births in our study is that women with twin pregnancies have greater GWG than women with singleton pregnancies and are more likely to experience risks such as preterm birth. The guidelines recommended by Institute of Medicine (IOM) for GWG in twin pregnancies are totally different from singleton pregnancies. Gestational age staging was based on last menstrual period date and available obstetric assessment using ultrasound. Women who delivered stillbirth or underwent termination of pregnancy (n = 16) were excluded. Those with missing data on weight and height in the beginning of pregnancy or invalid delivery weight (n = 53) were excluded. Mothers with pre-existing hypertension or diabetes, which are strong risk factors for preterm birth, were also excluded (n = 58). In total, 4274 women and 4274 infants were included in this study.

### Procedures

The clinical variables included: (1) General information: maternal age, education level, pre-pregnancy weight and height, delivery weight of pregnant women; (2) Gravidity, parity, maternal basic diseases, previous history of preterm birth; (3) Antenatal care visit, complications of current pregnancy, mode of conception; (4) Neonatal information: neonatal sex, gestational age.

Pre-pregnancy BMI was classified into four groups according to the 2009 IOM standards: underweight (< 18.5 kg/m^2^), normal weight (18.5–24.9 kg/m^2^), overweight (25–29.9 kg/m^2^), and obese (≥ 30 kg/m^2^)^8^.

Total GWG was obtained by subtracting pre-pregnancy weight from delivery weight. According to the 2009 IOM Guidelines, recommended GWG for underweight is 12.5–18 kg, normal weight is 11.5–16 kg, overweight is 7–11.5 kg, and obese is 5–9 kg^8^.

In our study, preterm birth was the main outcome, which was defined delivering less than 37 weeks. Slightly different from traditional classification system^29^, preterm birth was further subdivided into three groups in our study: extremely preterm birth (28–31 + 6 weeks’ gestation), very preterm birth (32–33 + 6 weeks’ gestation), and moderately preterm birth (34–36 + 6 weeks’ gestation).

### Statistical analysis

Maternal and neonatal demographic and clinical features are reported as frequency (%) or means (± SD). The rates of preterm birth according to population characteristics were calculated. Categorical variables were analyzed by chi-squared tests. Logistic regression models were performed to estimate the odds ratios (ORs) and 95% confidence interval (CI) for preterm birth and its subgroups. Pre-pregnancy BMI, maternal age, education levels, parity, gravidity, previous history of preterm birth, mode of conception, prenatal care, timing of initiation of prenatal care, pregnancy-induced hypertension, gestational diabetes mellitus and sex of infant were adjusted in our analyses. Otherwise, to further investigate the relationship between GWG and preterm birth, we did stratified analyses and explore the joint effect of pre-pregnancy BMI and maternal age. Two-sided p values less than 0.05 were considered significant. All statistical analyses were done with SPSS 20.0 software.

## Supplementary information

Supplementary Information

## Data Availability

The datasets generated during and/or analysed during the current study are available from the corresponding author on reasonable request.
