# Supplementary material for: Association between maternal gestational weight gain and preterm birth according to body mass index and maternal age in Quzhou, China
Source: Sci Rep. 2020 Sep 28;10:15863. doi: 10.1038/s41598-020-72949-w (PMC7522279; doi:10.1038/s41598-020-72949-w)
Supplement: Supplementary file 1 — Supplementary Information [file 41598_2020_72949_MOESM1_ESM.pdf]

Association between maternal gestational weight gain and preterm birth according to body mass index and maternal age in Quzhou, China

Ying Hu[1,2#], Qi Wu[1#], Luyang Han[1], Yuqing Zou[1,2], Die Hong[1], Jia Liu[1], Yuying Zhu[2], Qiumin Zhu[2], Danqing Chen[1], Lu Qi[3*], Zhaoxia Liang[1,3*]

[1] Obstetrical Department, Women's Hospital, School of Medicine, Zhejiang University, Hangzhou, China

[2] Quzhou Maternal and Child Health Hospital, Quzhou Maternal and Child Medical Association, Zhejiang University

[3] Department of Epidemiology, School of Public Health and Tropical Medicine, Tulane University, New Orleans, LA

[#] Ying Hu and Qi Wu contributed equally to this work

[*] Co-corresponding author

[*]**Correspondence to** Zhaoxia Liang. Obstetrical Department, Women's Hospital, School of Medicine, Zhejiang University, Hangzhou, China. 310006. E-mail: xiaozaizai@zju.edu.cn, Tel: +86571-87061501

Table S1. Prevalence of preterm birth in each GWG category.

	Low GWG					Adequate GWG					Excess GWG				
	N	n	n1	n2	n3	N	n	n1	n2	n3	N	n	n1	n2	n3
**Overall**	965	114 (11.8%)	83 (8.6%)	18 (1.9%)	13 (1.3%)	2135	150 (7.0%)	118 (5.5%)	21 (1.0%)	11 (0.5%)	1174	51 (4.3%)	49 (4.2%)	2 (0.2%)	0
**Age groups**															
**<20**	13	4 (30.8%)	2 (15.4%)	0	2 (15.4%)	17	4 (23.5%)	2 (11.8%)	0	2 (11.8%)	17	0	0	0	0
**20-29**	480	49 (10.2%)	37 (7.7%)	9 (1.9%)	3 (0.6%)	1158	76 (6.6%)	62 (5.4%)	9 (0.8%)	5 (0.4%)	688	20 (2.9%)	18 (2.6%)	2 (0.3%)	0
**30-34**	283	34 (12.0%)	22 (7.8%)	7 (2.5%)	5 (1.8%)	626	38 (6.1%)	32 (5.1%)	5 (0.8%)	1 (0.2%)	309	17 (5.5%)	17 (5.5%)	0	0
**≫35**	189	27 (14.3%)	22 (11.6%)	2 (1.1%)	3 (1.6%)	331	32 (9.7%)	22 (6.6%)	7 (2.1%)	3 (0.9%)	160	14 (8.8%)	14 (8.8%)	0	0
**Educational level**															
**Primary**	35	7 (20.0%)	5 (14.3%)	0	2 (5.7%)	41	5 (12.2%)	3 (7.3%)	1 (2.4%)	1 (2.4%)	20	1 (5.0%)	1 (5.0%)	0	0
**Secondary**	455	64 (14.1%)	44 (9.7%)	13 (2.9%)	7 (1.5%)	1014	71 (7.0%)	51 (5.0%)	11 (1.1%)	9 (0.9%)	620	32 (5.2%)	31 (5.0%)	1 (0.2%)	0
**College**	457	41 (9.0%)	33 (7.2%)	5 (1.1%)	3 (0.7%)	1037	73 (7.0%)	63 (6.1%)	9 (0.9%)	1 (0.1%)	514	17 (3.3%)	16 (3.1%)	1 (0.2%)	0
**Post-graduate**	18	2 (11.1%)	1 (5.5%)	0	1 (5.5%)	43	1 (2.3%)	1 (2.3%)	0	0	20	1 (5.0%)	1 (5.0%)	0	0
**Gravidity (before current pregnancy)**															
**0**	263	26 (9.9%)	22 (8.4%)	2 (0.8%)	2 (0.8%)	595	47 (7.9%)	38 (6.4%)	7 (1.2%)	2 (0.3%)	350	8 (2.3%)	6 (1.7%)	2 (0.6%)	0
**1-2**	481	59 (12.3%)	38 (7.9%)	13 (2.7%)	8 (1.7%)	1012	63 (6.2%)	46 (4.5%)	10 (1.0%)	7 (0.7%)	561	20 (3.6%)	20 (3.6%)	0	0
**≫3**	221	29 (13.1%)	23 (10.4%)	3 (1.4%)	3 (1.4%)	528	40 (7.6%)	34 (6.4%)	4 (0.8%)	2 (0.4%)	263	23 (8.7%)	23 (8.7%)	0	0
**Parity (before current pregnancy)**															
**Nullipara**	409	50 (12.2%)	36 (8.8%)	8 (2.0%)	6 (1.5%)	1034	78 (7.5%)	64 (6.2%)	10 (1.0%)	4 (0.4%)	646	14 (2.2%)	12 (1.9%)	2 (0.3%)	0
**Multipara**	556	64 (11.5%)	47 (8.5%)	10 (1.8%)	7 (1.3%)	1101	72 (6.5%)	54 (4.9%)	11 (1.0%)	7 (0.6%)	528	37 (7.0%)	37 (7.0%)	0	0
**Pre-pregnancy BMI(kg/m^2)**															

**Underweight**	190	15 (7.9%)	10 (5.3%)	3 (1.6%)	2 (1.1%)	371	14 (3.8%)	12 (3.2%)	1 (0.3%)	1 (0.3%)	134	6 (4.5%)	6 (4.5%)	0	0
**Normal**	739	93 (12.6%)	67 (9.1%)	15 (2.0%)	11 (1.5%)	1609	117 (7.3%)	96 (6.0%)	15 (0.9%)	6 (0.4%)	785	28 (3.6%)	28 (3.6%)	0	0
**Overweight and Obese**	36	6 (16.7%)	6 (16.7%)	0	0	155	19 (12.3%)	10 (6.5%)	5 (3.2%)	4 (2.6%)	255	17 (6.7%)	15 (5.9%)	2 (0.8%)	0
**Previous history of preterm birth**															
**Yes**	34	8 (23.5%)	5 (14.7%)	1 (2.9%)	2 (5.9%)	59	12 (20.3%)	8 (13.6%)	2 (3.4%)	2 (3.4%)	26	8 (30.8%)	8 (30.8%)	0	0
**No**	522	56 (10.7%)	42 (8.0%)	9 (1.7%)	5 (1.0%)	1042	60 (5.8%)	46 (4.4%)	9 (0.9%)	5 (0.5%)	502	29 (5.8%)	29 (5.8%)	0	0
**Nullipara**	409	50 (12.2%)	36 (8.8%)	8 (2.0%)	6 (1.5%)	1034	78 (7.5%)	64 (6.2%)	10 (1.0%)	4 (0.4%)	646	14 (2.2%)	12 (1.9%)	2 (0.3%)	0
**Mode of conception**															
**Nature**	946	112 (11.8%)	82 (8.7%)	18 (1.9%)	12 (1.3%)	2079	141 (6.8%)	112 (5.4%)	19 (0.9%)	10 (0.5%)	1136	48 (4.2%)	46 (4.0%)	2 (0.2%)	0
**ART**	19	2 (10.5%)	1 (5.3%)	0	1 (5.3%)	56	9 (16.1%)	6 (10.7%)	2 (3.6%)	1 (1.8%)	38	3 (7.9%)	3 (7.9%)	0	0
**Prenatal care**															
**none**	10	1 (10.0%)	0	1 (10.0%)	0	11	3 (27.2%)	1 (9.1%)	0	1 (9.1%)	4	0	0	0	0
**Our hospital**	690	66 (9.6%)	54 (7.8%)	5 (0.7%)	7 (1.0%)	1599	93 (5.8%)	79 (4.9%)	10 (0.6%)	4 (0.3%)	896	40 (4.5%)	39 (4.4%)	1 (0.1%)	0
**Other hospitals**	265	47 (17.7%)	29 (10.9%)	12 (4.5%)	6 (2.3%)	525	54 (10.3%)	37 (7.0%)	11 (2.1%)	6 (1.1%)	274	11 (4.0%)	10 (3.6%)	1 (0.4%)	0
**Timing of initiation of prenatal care**															
**1st-3rd month**	861	102 (11.8%)	74 (8.6%)	16 (1.9%)	12 (1.4%)	1927	130 (6.7%)	103 (5.3%)	19 (1.0%)	8 (0.4%)	1045	47 (4.5%)	45 (4.3%)	2 (0.2%)	0
**4th-6th month**	88	11 (12.5%)	9 (10.2%)	1 (1.1%)	1 (1.1%)	185	16 (8.6%)	12 (6.5%)	2 (1.1%)	2 (1.1%)	120	3 (2.5%)	3 (2.5%)	0	0
**7th to final month**	6	0	0	0	0	12	1 (8.3%)	1 (8.3%)	0	0	5	1 (20.0%)	1 (20.0%)	0	0
**none**	10	1 (10.0%)	0	1 (10.0%)	0	11	3 (27.2%)	2 (9.1%)	0	1 (9.1%)	4	0	0	0	0
**Pregnancy-induced hypertension**	17	5 (29.4%)	3 (17.6%)	1 (5.9%)	1 (5.9%)	58	11 (19.0%)	8 (13.8%)	3 (5.2%)	0	51	6 (11.8%)	5 (9.8%)	1 (2.0%)	0

**Gestational diabetes mellitus**	265	25 (9.4%)	17 (6.4%)	4 (16.0%)	4 (16.0%)	428	32 (7.5%)	24 (5.6%)	7 (1.6%)	1 (0.2%)	229	13 (5.7%)	12 (5.2%)	1 (0.4%)	0
**Infant Sex**															
**Male**	481	60 (12.5%)	43 (8.9%)	11 (2.3%)	6 (1.2%)	1140	96 (8.4%)	74 (6.5%)	14 (1.2%)	8 (0.7%)	599	29 (4.8%)	28 (4.7%)	1 (0.1%)	0
**Female**	484	54 (11.2%)	40 (8.3%)	7 (1.4%)	7 (1.4%)	995	54 (5.4%)	44 (4.4%)	7 (0.7%)	3 (0.3%)	575	22 (3.8%)	21 (3.7%)	1 (0.1%)	0

Data were n/N (%). N means total births records; n means preterm births records; n1 means moderately preterm births records; n2 means very preterm births records; n3 means extremely preterm births records.
